# Durability of Ultem 9085 in Marine Environments: A Consideration in Fused Filament Fabrication of Structural Components

**DOI:** 10.3390/polym16030350

**Published:** 2024-01-28

**Authors:** Xiong (Julia) Wang, Carly Travis, Mark T. Sorna, Dwayne Arola

**Affiliations:** 1 Department of Materials Science and Engineering, University of Washington, Box 352120, Seattle, WA 98195-2120, USA; xironw@uw.edu (X.W.);; 2 Naval Undersea Warfare Center, Keyport, WA 98345-7610, USA; mark.t.sorna.civ@us.navy.mil; 3 Department of Mechanical Engineering, University of Washington, Seattle, WA 98195-2120, USA

**Keywords:** accelerated aging, fused filament fabrication, hydrolysis, Ultem 9085

## Abstract

The long-term durability of polymer components produced by additive manufacturing (AM) in marine conditions is poorly understood. Here, fused filament fabrication (FFF) of Ultem 9085 was conducted and accelerated aging was performed. Two printing orientations (−45/45° and 0/90°) and two sample types (ASTM D638 Type 1 and Type 4) were produced and subjected to accelerated aging in either seawater or air. Results from tensile tests showed that the elastic modulus, yield strength and ultimate tensile strength increased after seawater aging, whereas the elongation to failure decreased. Results of thermogravimetric analysis (TGA) and derivative–TGA curves indicated that hydrolysis occurred after seawater exposure to the polycarbonate (PC) component and changes in structure or hydrogen bonds formed in the polyetherimide (PEI) component. Differential scanning calorimetry showed that physical aging occurred after short exposure periods and low temperature. Longer exposures and higher temperatures resulted in increasing plasticization by water and scission of the PC molecules. Results from Raman suggest that hydrolysis of the PC occurred, with a reduction in free volume produced by physical aging or hydrogen bonding with water molecules. These results highlight that Ultem 9085 is susceptible to degradation in marine environments, and there are two primary mechanisms, including physical and chemical aging. Their specific contribution is highly sensitive to the aging temperature and require careful selection in accelerated aging evaluations.

## 1. Introduction

Additive manufacturing (AM) represents a suite of novel processes for creating three-dimensional physical objects directly from computer-aided design (CAD) models via layer-by-layer construction [1]. Also commonly regarded as 3D printing, AM is considered capable of creating structures with nearly unlimited complexity, with a substantial reduction in time from conception to finished product in comparison with traditional manufacturing processes. Consequently, it has received substantial attention in the last decade. Specific AM processes are being adopted in various industrial sectors, including the aerospace, automotive and medical industries [2].

Fused filament fabrication (FFF) is one of the most common techniques for AM and is used for printing components of thermoplastic polymers, including prototypes and functional end-use parts. In FFF, a polymer filament is extruded from a heated nozzle and deposited onto the printing bed or a previous printed layer in a series of continuous lines with specific orientation [3]. The mechanical properties of components that are produced by FFF depend on the printing parameters, which are optimized to maximize the part quality, the microstructure and overall printing process economy [4,5]. The process physics in FFF involves melting and solidification of the polymer, which can give rise to localized porosity and anisotropic mechanical properties in parts [6]. Nevertheless, the affordability of FFF printing systems and the tremendously large number of feedstock materials available for FFF make it one of the most ubiquitous AM processes overall [4,5,6].

Underwater and marine structures are an interesting niche application for AM, such as autonomous underwater vehicles, micro underwater vehicles and unmanned surface vehicles [3]. When compared with traditional manufacturing methods, AM could enable these systems to be developed with lower cost and minimal time from conception to final product, substantially increasing the response readiness. However, these applications bring new challenges and concerns, including the durability of 3D-printed products under environmental threats posed by a marine environment [4,7]. Applications that require durability must address the multiple sources for degradation, including exposure to ultraviolet (UV) light, pressure cycles, chemical attack and the potential for hydrolytic damage [4,8,9,10]. As such, if the components are intended for use in mission-critical applications, these sources of marine environment degradation and their ramifications must be understood. 

Polymer feedstock materials for FFF that are intended for applications in marine environments should possess the appropriate resistance to degradation [11,12]. However, the data concerning this aspect of performance are scant. Ultem 9085 is an advanced engineering polymer for FFF that is adopted for applications that require outstanding mechanical and thermal properties and good chemical resistance [6]. It is recognized for its flame retardancy, low toxicity, dimension stability and retention of strength at high temperatures. As such, it is sought for marine, aerospace and automotive applications, especially where low-weight and high-strength materials are required [13]. In addition, its high environmental resistance and high strength-to-weight ratio characteristics make it a promising candidate for the renewable energy and turbomachinery industries [14,15]. Ultem 9085 is a blend of polyetherimide (PEI) with polycarbonate (PC) copolymer, which is added to improve the printability of PEI [5]. Based on the importance of its structural behavior, the mechanical properties of Ultem 9085 have been assessed after FFF through tensile, fatigue, compression and flexural testing [16,17,18,19,20,21,22,23]. Nevertheless, the response to threats related to marine conditions have received limited attention. 

PEI is an amorphous engineering thermoplastic with excellent performance. The qualities of PEI include high strength, high stiffness and capacity for high-temperature resistance [24]. There are also limitations to PEI, such as its comparatively high cost and instability in chlorinated solvents. Polycarbonate is an amorphous engineering thermoplastic that is widely used to blend with other materials because of its desirable strength and stiffness, as well as its outstanding compatibility with a series of polymers [25]. However, PC can undergo degradation under exposure to bases and moisture, especially at elevated temperatures [26]. Hence, there are qualities of PEI and PC that suggest that Ultem 9085 could be susceptible to degradation under prolonged exposure to marine conditions. 

In recent years, there has been considerable research focused on printing conditions to improve the mechanical performance of 3D-printed Ultem 9085. Shelton et al. developed a chamber to control the printing environment temperature during FFF of Ultem 9085 [27]. It was found that the capacity for inelastic deformation and post-neck yielding increased with build chamber temperature, which had a large effect on the mechanical properties [27]. Zaldivar et al. evaluated the thermal and mechanical behavior of 3D-printed Ultem 9085 with respect to build orientations [6]. Their results demonstrated that the mechanical properties of the printed samples were heavily dependent on the printing orientations [6]. That group also investigated the influence of filament moisture content on the mechanical properties of the printed material, finding that an increase in voids and poor filament orientation arose with increasing moisture content, which were detrimental to the performance [5]. Post-processing of FFF prints can be applied for further improvements. For instance, atmospheric plasma treatment, controlled cooling and post-process annealing have also been explored for enhancing the mechanical performance of Ultem 9085 following FFF [28,29,30,31]. 

Despite the importance of degradation in structure and properties posed by marine applications, only limited assessment of the environmental effects on 3D-printed Ultem 9085 material has been reported. Padovano et al. conducted the thermal aging test on Ultem 9085 by treating samples with high temperature, cyclic climate change and thermal shock [21]. They concluded that the progressive variation in temperature or constant high temperature did not cause significant degradation to the mechanical properties. However, sudden cooling from a high temperature could degrade the mechanical properties of Ultem 9085. A previous study exposed Ultem 9085 to various media and air and water at temperatures from −60 °C to 160 °C (which is highly relevant to industrial applications) [32]. While water immersion for up to 52 weeks had negligible effects on its mechanical performance, exposure to an alkaline cleaner caused degradation after only 1 week of exposure [32]. Gallagher explored the degradation of 3D-printed Ultem 9085 to UV exposure, elevated temperatures and vacuum conditions for up to three months [33]. Results showed that the UV and vacuum environment at elevated temperature caused stiffening and strengthening of Ultem 9085 but a reduction in the strain to fracture [15]. Considerations related to changes in durability with exposure to seawater are just now appearing [15,34].

Environmental degradation over an exposure period can be regarded as aging. In general, there are two categories of “aging” that can happen to polymers, including chemical aging and physical aging, both of which can affect the physical properties [35]. Chemical aging is generally related to the modification of the polymer chain and can result in chain scission or oxidation [35]. It can happen in both processing and applications, where elevated temperatures, air, light, moisture and living organisms and other factors exist [36]. Chemical aging is irreversible and usually causes degradation of the microstructure and mechanical properties of the polymers. Hydrolysis is a common form of chemical aging that involves the breakage of polymer bonds by water molecules, which can occur in marine environments and cause degradation of the performance of polymers. 

Contrary to chemical aging, physical aging is a reversible process driven by the non-equilibrium state of polymer chains induced by rapid cooling of the molten polymer in the manufacturing process [35]. During a sudden drop in temperature, the polymer chains do not have ample time to relax and remain in a distorted configuration compared with the chain distribution in an equilibrium state. This phenomenon is especially common in amorphous polymers where the chains pursue densification in an equilibrium state [37]. The reduction in free volume can increase the intermolecular attraction of the molecules, which will increase the strength and stiffness at a macro level but cause lower ductility. Physical aging is enabled when the environmental temperature exceeds that required for mobility of the side chains and localized groups. The propensity and rate of physical aging increases with exposure temperature and its proximity to the glass transition temperature (Tg) [37]. 

Accelerated aging is a common experimental approach that is adopted to assess the long-term degradation of a material by conducting short-term tests. These are performed using selected conditions that typically involve substantially more aggressive levels of exposure than in natural exposure [38]. The purpose of acceleration is to reduce the required testing time. Polymers, in particular, are susceptible to degradation in a variety of conditions, including exposure to elevated temperatures, moisture, chemicals, ultraviolet light, oxygen, etc. In accelerated aging, the experimental conditions should emulate those of the application to develop data through the responses that are most appropriate for predicting performance. In fact, this is a common approach to support the development of reliability and lifetime prediction models [38]. For example, Davis and Evrard applied accelerating aging to build an Arrhenius model for the degradation of polyurethane in marine environments [39]. Similarly, Tocháček and Vrátníčková utilized accelerated UV aging to build a lifetime prediction model for polypropylene and its copolymers in outdoor exposures [40]. Using results from accelerated thermal and hydrothermal aging, Bergaliyeva et al. simulated the mechanical properties of 3D-printed polylactic acid after 1.5–2.5 years of service [41]. 

It is important to highlight that accelerated aging evaluations employed to assess the durability of polymers under specific threats (ultraviolet light, moisture, chemicals, etc.) are potentially controversial. The acceleration of aging could illicit mechanisms of degradation or combinations of mechanisms that are quite different from those that are experienced in the natural environment-intended applications [42]. As such, it is important to treat the results and their applications with caution and understand the potential limitations. Nevertheless, accelerated aging can serve as an effective measure of sensitivity to degradation and can be helpful for rating the durability of polymeric materials.

The outstanding properties of Ultem 9085 make it a highly viable material for marine applications. Yet, the durability of this material under prolonged marine environment exposure is relatively unknown. As a result, the objective of this investigation is to evaluate the degradation resistance of 3D-printed Ultem 9085 produced by FFF under exposure to seawater. To achieve this objective, accelerated aging tests were performed in a standardized marine environment. In this investigation, an accelerated aging approach was adopted to evaluate the resistance of 3D-printed Ultem 9085 to degradation under prolonged exposure to seawater. The quality of the printed material was evaluated by optical profilometry, tensile testing and μCT scanning before and after exposure to the seawater environments. Here, results of these accelerated aging evaluations are presented, the contributing mechanisms are assessed and the feasibility of applying accelerating aging tests for Ultem 9085 is discussed.

## 2. Materials and Methods

### 2.1. Materials and Printing

**Figure 1:**
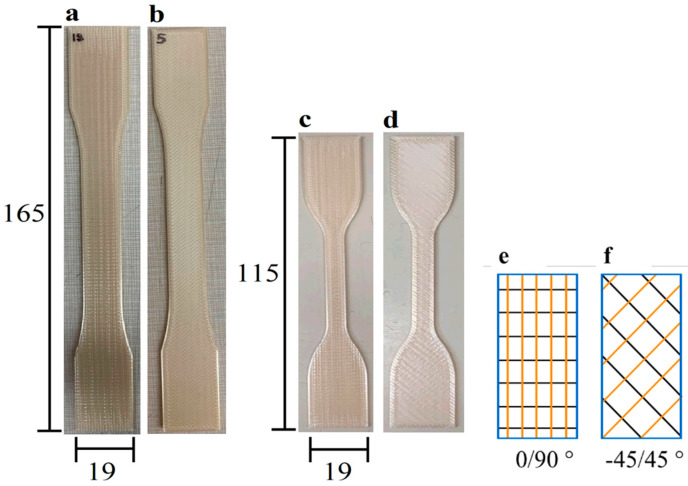
Details regarding the 3D-printed Ultem 9085 tensile bars. (**a**) Type 1, 0/90° orientation; (**b**) Type 1, −45/45° orientation; (**c**) Type 4, −45/45° orientation; (**d**) Type 4, 0/90° orientation; (**e**,**f**) illustration of printing orientations (unit: mm).

The material evaluated in this investigation was Ultem 9085, which was acquired from Stratasys by the Naval Undersea Warfare Center (NUWC, Keyport, WA, USA). Tensile specimens were printed by fused filament fabrication (FFF) using a Fortus 900MC from Stratasys. All printing was performed using the default settings of the machine for Ultem 9085 (which included a layer thickness of 0.254 mm) and an aerospace nozzle tip. The remaining parameters associated with the default conditions of printing Ultem 9085 with this printer are proprietary. To examine the potential influence of printing orientation, two groups of samples were prepared with infill orientations of −45/45° and 0/90°, as shown in Figure 1. These two orientations were included because they are the most common orientations chosen for printing of components. Furthermore, an infill percentage of 100% was used to reduce the relative effects of porosity on the mechanical behavior, and that is the intended form of application. 

The standard ASTM D638 is widely used for evaluating the mechanical properties of polymers and assessing the influence of manufacturing methods on the mechanical behavior [43]. There is presently no additional guideline that defines how the mechanical properties of products manufactured by FFF should be evaluated. Therefore, Type 1 and Type 4 tensile specimens were printed with a 1.59 mm thickness by FFF according to the geometry described in ASTM D638 [44]. A total of 120 specimens were prepared to assess the effects of marine exposure, including 20 specimens with Type 1 geometry for each of the 2 infill orientations and 40 specimens with the Type 4 geometry for each of the orientations (2 × 20 + 2 × 40 = 120).

### 2.2. Accelerated Aging

The accelerated aging testing in seawater was performed with multiple digital thermostatic water baths (DK-2000-IIIL-4, Faithful, Huanghua, Cangzhou, China) and with a bath volume of approximately 12 L. The marine environment for aging was designed after prior-published work [39], utilizing standardized artificial seawater that was prepared in accordance with ASTM D114 [45]. A standard seawater sample was chosen as the aging medium (rather than an arbitrary natural seawater sample) to adhere to available guidelines for assessing the importance of seawater exposure to polymer durability and to ensure that the methods could be easily repeated by other investigators. The aging conditions used for evaluating the Ultem 9085 samples are described in Table 1. Ten tensile coupons were immersed within the standardized seawater baths at ambient pressure and at temperatures of 50 °C, 70 °C and 90 °C. It is important to note that these elevated temperatures were not indicative of the application temperatures envisioned for this material and that the conditions did not replicate or mimic the natural service conditions. They were chosen specifically to promote more rapid degradation if the polymer was susceptible while also remaining below critical temperatures for this material. Here, the selected temperature range for this evaluation was chosen because it is a common range used in performing accelerated aging of polymers and it is below the glass transition temperature of Ultem 9085 (186 °C) [38,39]. The specimens were removed from the bath after an exposure period of either 2 or 4 weeks and tensile tests were conducted to evaluate the degradation thereafter. 

Due to the lower number of Type 1 0/90° samples, the aging conditions applied to those samples included 50 °C for 14 days and 90 °C for 14 and 28 days. Pilot experiments were performed on extruded polyurethane sheet as a reference material to confirm the validity of the accelerated aging approach. According to the results of the aging evaluations in seawater, it was identified that complimentary air aging experiments should be conducted to characterize the physical aging that occurs during the aging period at elevated temperatures. Therefore, accelerated aging evaluations were conducted with Ultem 9085 in air under the temperatures and durations outlined in Table 1. These experiments were conducted on additional specimens printed with the two different infill orientations.

### 2.3. Water Uptake

The uptake of water is an important concern for undersea and marine structures. Therefore, the change in weight of the samples was measured before and after the exposure periods with an electric balance (Model JM-303, RESHY, Huzhou, Zhexing, China) with ±0.001 g precision. After the aging experiments and prior to the weight measurements, excess moisture on the surface of the samples was blotted dry by tissue paper. The extent of water uptake percentage (w_wu_%) was estimated by taking weight measurements of 5 samples of each sample configuration before (w_o_) and after (w_e_) of the exposure period according to:

(1) $$w_{\mathrm{wu}}\%=\frac{w_{e}-w_{0}}{w_{0}}\times 100\%$$

### 2.4. Profilometry

Profilometry was performed to characterize the surface topography of the printed samples using an optical profilometer (VR-3100 3D, KEYENCE, Osaka, Japan). One sample of each geometry was randomly selected to characterize the surface topography prior to aging. To inspect the surfaces for characteristics that could contribute to the mechanical behavior, topographical images were taken on the printed side of the gauge section, grip section and transition areas of the specimens at 12× magnification. The average roughness (Ra) and ten-point roughness (Rz) of the specimens were estimated using images obtained at higher magnification (×40) on the printed side and the edge of the gauge section using a traverse length of 5.6 mm. The Ra represents the mathematical average deviation of the surface from the mean line over the traverse length, and the Rz is the average deviation of the five highest peaks and valleys from the mean line. The roughness parameters were calculated using the multiline roughness software that is provided with the instrument. An upper cut-off (lc) and lower cut-off (ls) of 0.08 µm and 25 µm were used in treating the height distribution, respectively.

### 2.5. Microcomputed Tomography

To understand water diffusion within the microstructure and its accumulation, microcomputed tomography (µCT) scans were performed on virgin printed samples using a commercial system (X5000, North Star Imaging, Rogers, MN, USA). The potential volume for absorbed liquid in the voids was computed through the areal porosity and the length of the voids. A 3D model for the Type 4 specimen was built by SOLIDWORKS (2020, Dassault Systèmes SOLIDWORKS Corp, Waltham, MA, USA) based on the ASTM D638 standard to estimate the volume of the specimen (2.557 cm^3^). The weight of the potential water uptake was calculated by multiplying the volume of the specimens (V), average areal porosity of the specimen (A%) and density of water (ρ) (assumed to be 1 g/cm^3^). The approximate weight of specimens prior to aging (w_s_) was 2.6 g. Based on those values, the theoretical maximum water uptake range based on filling of the pores (w_t_%) was obtained according to the formula

(2) $$w_{t}\%=\frac{V\times A\times \rho }{W_{s}}\times 100\%$$

### 2.6. Tensile Testing

Tensile testing of the printed specimens was performed in ambient conditions using a universal testing system (E1000 Dynamic Test instrument, Instron, Norwood, MA, USA) with a 2 kN load cell. The loading was conducted in displacement control at a rate of 5 mm/min according to the ASTM D638 standard guidance for rigid polymers [44]. Four samples each of Type 1 and Type 4 were tested in the unaged condition for each of the two orientations to establish the control responses. Five samples from each aged condition were tested and the stress–strain responses were used in estimating the elastic modulus (E), yield strength (YS), ultimate tensile strength (UTS) and strain at failure. According to guidelines outlined by ASTM D638, the test was only considered valid if failure occurred within the gauge section of the sample; results for samples that failed outside of the gauge section were disregarded. For each property assessed, the average, standard deviation and coefficient of variation were determined. The yield strength was determined from the 0.2% offset method. Two sample *t*-tests with unequal variance were applied to identify significant differences in the mechanical properties between the aged and unaged specimens. Significant differences were identified by *p* ≤ 0.05.

### 2.7. Thermogravimetric Analysis and Differential Scanning Calorimetry

Thermogravimetric analysis (TGA) was performed on the Ultem samples before and after aging using a commercial instrument (TGA Q50, TA Instruments, New Castle, DE, USA). Approximately 5 mg was obtained from the sample of interest and heated within a nitrogen atmosphere from ambient environment temperature to 800 °C at a 10 °C/min heating rate. Conventional TGA as well as D-TGA (derivative TGA) curves were prepared to analyze the changes in molecular conformation and potential degradation that resulted from aging.

Differential scanning calorimetry (DSC) was also performed to estimate the glass transition temperature (Tg), relaxation enthalpy (*δ*_H_) and peak temperature (Tp) of the Ultem samples using a commercial instrument (Q20, TA instruments, New Castle, DE, USA). Samples of approximately 3 mg were sealed in a Tzero aluminum pan with a Tzero aluminum lid. The sealed crucible was heated at a rate of 10 °C/min in a nitrogen environment. The maximum heating temperature was analyzed from the TGA curves up to 300 °C for the samples.

### 2.8. Raman Spectroscopy

To evaluate the molecular structure for the untreated and treated samples, Raman spectroscopy was performed using a commercial instrument (inVia Raman microscope, Renishaw, Wotton-under-Edge, UK). Raman spectra were generated using a 784 nm laser source with 10 s exposure on the surface of the sample with a 50× objective. The spectra were obtained over wavelengths from 100 to 3200 cm^−1^. Samples of material for Raman spectroscopy were sectioned from the grip sections of the fractured samples. Baseline correction, normalization and smoothing of the Raman spectra were achieved by data analysis software (Origin, OriginLab, Northampton, MA, USA). In addition, WiRE software (Version 5.2) was used to acquire and compare the Raman spectra for Ultem 1000 to the spectra for PC.

## 3. Results

### 3.1. Mechanical Properties 

**Figure 2:**
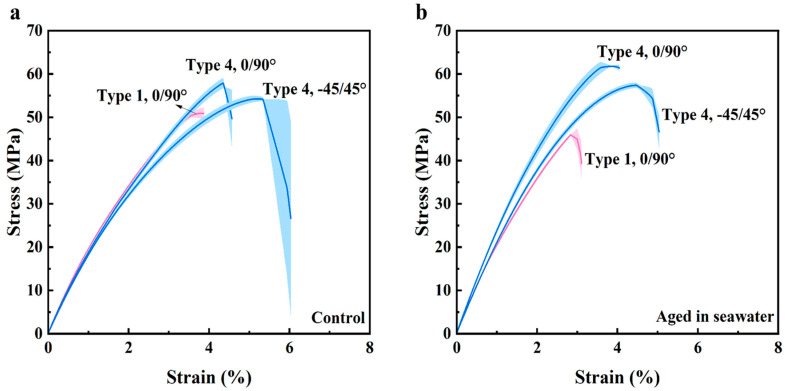
Stress–strain response of Ultem 9085 at different conditions. (**a**) Unaged samples; (**b**) samples submerged in seawater at 90 °C for 28 days.

**Figure 3:**
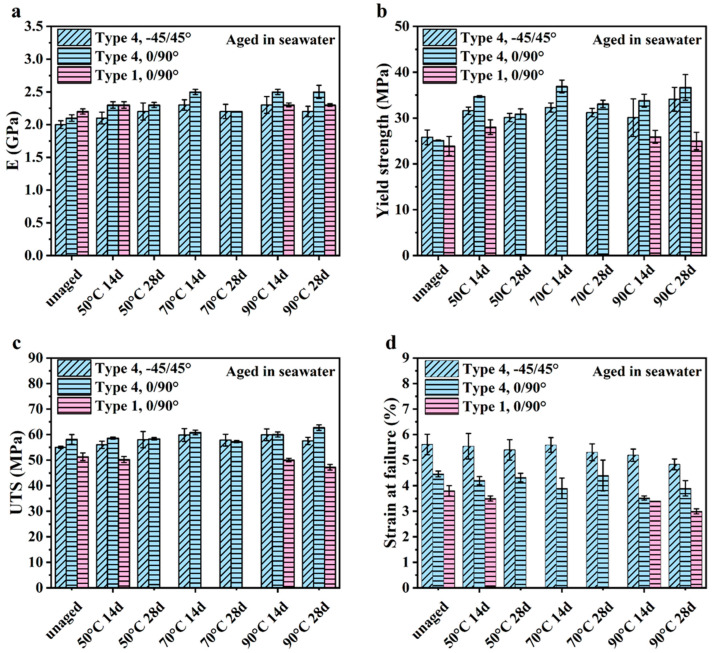
Mechanical properties of the unaged and aged Ultem 9085 samples in seawater determined by their strain–stress curves. (**a**) Elastic modulus (E); (**b**) yield strength; (**c**) ultimate tensile strength (UTS); (**d**) strain at failure. Note: results for the Type 1 −45/45° group were not valid due to failure outside of the gauge section and are not included in the reported properties.

Representative stress–strain curves for the unaged control samples and after accelerated aging in seawater are shown in Figure 2a and Figure 2b, respectively. The aging condition in Figure 2b consists of aging in seawater at 90 °C for 28 days. Results in this figure are presented for both the Type 1 and Type 4 specimens and the two infill orientations. The mechanical properties were determined from these curves and are summarized in Figure 3. The complete data set is listed in the Supplemental Information (SI, Tables S1–S4). It is important to note that, due to printing anomalies and stress concentrations in the transition areas associated with the infill, some of the specimens failed prematurely and outside of the gauge section (Figure 2). As such, the tensile data for those specimens were excluded when estimating the mechanical properties.

The elastic modulus of the samples in Figure 3a revealed that there was an increase in the stiffness of the samples after aging in the artificial seawater regardless of temperature. The yield strength distribution for the control and aged conditions is presented in Figure 3b and shows that the yield strength increased with aging and with respect to the control. Similarly, according to the tensile responses in Figure 3c, the UTS also increased with aging, although not as markedly as the increase in yield strength. The UTS of the Type 1 0/90° samples was lower than that of the UTS of Type 4 0/90° samples in the control condition, which could be attributed to differences in the porosity between the two sample types. Noteworthy, the UTS of the Type 4 samples with −45/45° increased after aging in seawater at all the elevated temperatures with respect to the control. However, the UTS of the Type 4 −45/45° and 0/90° infill samples did not exhibit a distinct trend with increasing temperature or exposure time. For the −45/45° printing orientation, the UTS decreased with increasing exposure time at both 70 and 90 °C. And, for the samples with 0/90° infill at 50 °C and 70 °C, there was a decrease in UTS with extended aging time. Unique from strength, Figure 3d shows that accelerated aging caused a decrease in the strain to failure of the samples in the seawater environment.

Detailed results for the Student’s *t*-test of the mechanical properties are listed in Tables S1–S4. It is noteworthy that all samples underwent a significant (*p* ≤ 0.05) increase in stiffness (reflected by E), except for the Type 1 0/90° specimens aged at 50 °C for 14 days. Only the yield strength of the Type 1 0/90° specimens and Type 4 −45/45° specimens exposed at 90 °C for 14 days did not show a change compared with the control specimens. Regarding UTS, aging at 70 °C for 14 days and at 90 °C had a significant effect on the Type 4 −45/45° samples. However, for the Type 1 0/90° and Type 4 0/90° samples, only aging at 90 °C for 28 days caused a significant change in the UTS of the 3D-printed material. The strain at failure decreased significantly for Type 4 0/90° after the aging at 90 °C for 14 days. At 90 °C, the elongation to failure of both the Type 4 −45/45° and Type 1 0/90° specimens underwent significant decreases after 28 days of accelerated aging.

The coefficient of variation (COV) for the Ultem 9085 samples is summarized in Table S5. Regarding the mechanical properties, the Type 4 −45/45° specimens had the largest COV overall in E, which increased after exposure to the marine environment at elevated temperatures. Likewise, the Type 4 −45/45° specimens had the highest COV in yield strength and UTS. Aging at 90 °C induced a large COV in yield strength for both Type 4 −45/45° and Type 4 0/90° samples, whereas aging at 50 °C with a longer exposure time caused higher COV in the UTS of Type 4 −45/45° samples. The COV for strain at failure increased the most for Type 4 0/90° samples submerged in 70 °C seawater and for Type 4 −45/45° samples submerged in 50 °C seawater. Overall, the Type 4 0/90° samples displayed the greatest COV in strain at failure.

In summary, the E and the yield strength for the 3D-printed Ultem 9085 materials increased significantly under the accelerating aging conditions. The UTS for this material did not increase significantly with aging, except for the longer exposure time (28 days) in seawater at 90 °C. The strain at failure for the Ultem 9085 material underwent a reduction with accelerated aging, but only exposure at 90 °C caused a significant decrease.

### 3.2. Failure Characteristics and Contributions 

**Figure 4:**
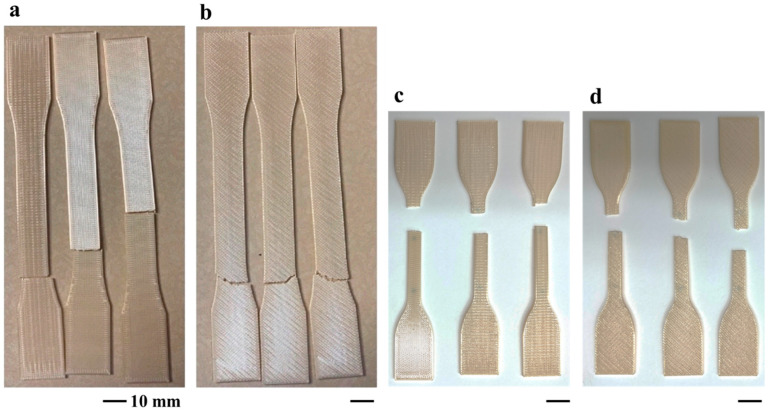
Tested unaged 3D-printed Ultem 9085 samples. (**a**) Type 1, 0/90° orientation; (**b**) Type 1, −45/45° orientation; (**c**) Type 4, 0/90° orientation; (**d**) Type 4, −45/45° orientation.

Figure 2 presents images from representative specimens after tensile testing to failure. Specifically, selected Type I samples with 0/90° and −45/45° orientations are presented in Figure 4a and Figure 4b, respectively, and corresponding Type 4 samples are shown in Figure 4c and Figure 4d, respectively. All the Type 1 samples with −45/45° infill orientation failed within the transition region between the gauge and grip section, which invalidated the mechanical testing results, whereas the 0/90° samples failed inside the gauge section and at the transition region. For the Type 4 geometry, some of the 0/90° samples broke at the transition from the grip and gauge sections, but notably fewer than occurred for the Type I geometry; none of the 45/45° infill orientation failed outside the gauge section. The documented locations of failure were used to categorize the results and censor the tensile testing response used when estimating the mechanical properties. All results related to failures that did not occur within the gauge section were considered invalid and were not used when evaluating the mechanical properties presented in Figure 3. 

**Figure 5:**
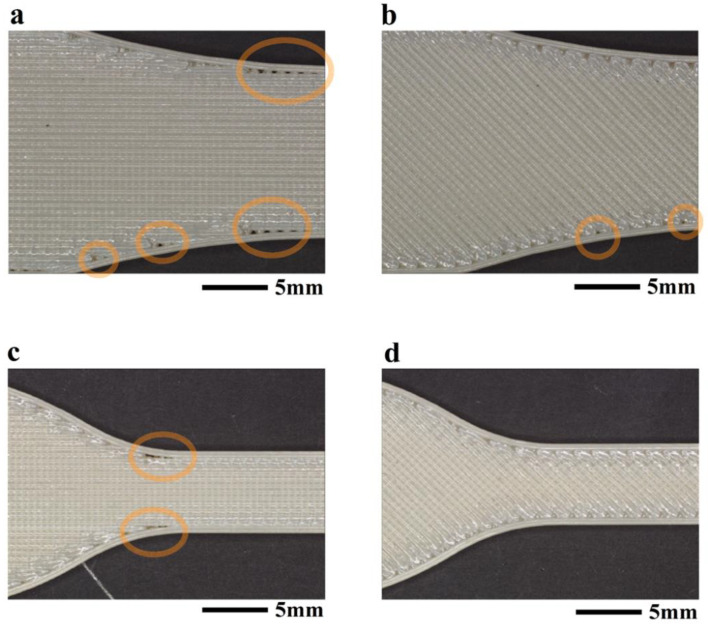
Microscopy images for the Ultem 9085 samples obtained from the optical profilometer. (**a**) Type 1, 0/90° orientation; (**b**) Type 1, −45/45° orientation; (**c**) Type 4, 0/90° orientation; (**d**) Type 4, −45/45° orientation. The yellow circles outline regions of voids at the boundary of contours.

Potential contributions to failure in the transition region of the tensile specimens were explored through optical evaluations and profilometry. All the sample configurations exhibited voids in the transition areas, as highlighted in Figure 5, except for the Type 4 samples with −45/45° infill, as evident in Figure 5d. For both the Type 1 and Type 4 samples, voids were identified in the transition areas between the contour and infill, as evident in Figure 5a–c. Indeed, previous studies have commented on how the infill path used for the printing of the ASTM D638 specimens can lead to stress concentrations in the transition region and contribute to the failure process [46,47,48]. 

**Figure 6:**
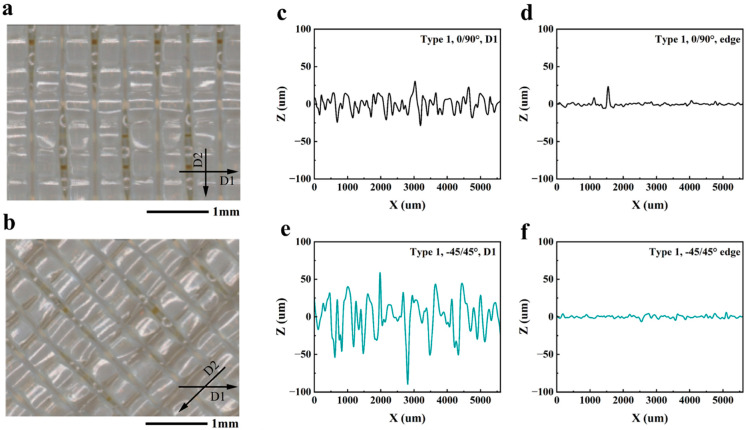
Optical images of the gauge section of the tensile samples with higher magnification and the roughness profile of the unaged Type 1 samples. (**a**) 0/90° printing orientation samples. D1: parallel to the contour, D2: perpendicular to the infill filament. (**b**) −45/45° printing orientation samples. D1: parallel to the contour, D2: perpendicular to the infill filament. (**c**) 0/90° orientation, D1; (**d**) 0/90° orientation, edge; (**e**) −45/45° orientation, D1; (**f**) −45/45° orientation, edge.

To assess the infill distribution and other aspects of the surface topography that could contribute to failures, the surface texture of the printed samples was evaluated. Profiles were obtained from the gauge section surface (Figure 6) and from along the edge. Two different orientations of analysis were applied to the specimen faces, as shown in Figure 6a,b. Representative surface roughness profiles taken parallel to the longitudinal axis and (direction of uniaxial tension (Direction 1)) are presented in Figure 6c,e for the 0/90° and −45/45° samples, respectively. Results of the roughness measurements are summarized in Table 2. As evident from these measurements, the samples with −45/45° infill orientation exhibited higher surface roughness in Direction 1, which resulted from gaps between the discrete infill tracks and lower infill density, as evident in Figure 6e. As evident from the surface roughness measurements in Table 2, both the Ra and Rz values of the edges were substantially lower than for the faces, which resulted from the smaller peak-to-valley height variations along the contours 

.

**Figure 7:**
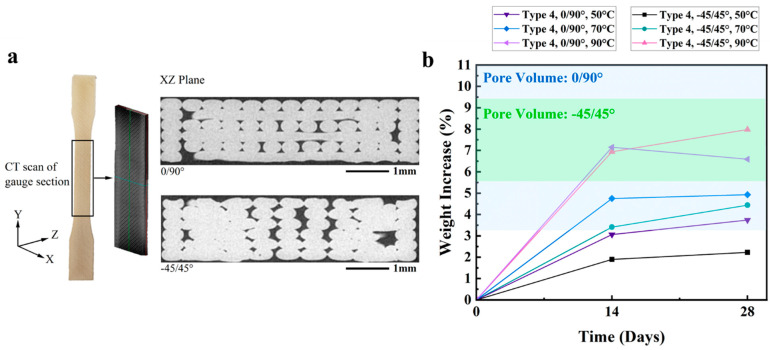
Details of the porosity. (**a**) Type 4 Ultem 9085 dog-bone CT scan result and the cross-section pictures. (**b**) Water uptake for Type 4 samples with respect to printing orientation and seawater temperature.

The porosity in the printed specimens was evaluated from the μCT scans obtained in the transition areas, as shown in Figure 7a. Utilizing the areal porosity estimates in the XZ plane, the maximum water uptake was estimated according to Equation (1) and results are listed in Table 3 as a function of specimen type and infill pattern. Overall, the samples with 0/90° orientation had greater porosity and water uptake than the −45/45° orientation samples. Figure 7b compares the measured water absorption of the samples at different temperatures after 14 and 28 days. For the Type 4 samples, the weight change caused by water inside the pores was estimated from the areal porosity using Equation (1) and is highlighted in the diagram. It is important to note that there appeared to be an increase in the extent of water update and weight increase with aging time and temperature. However, the rate of increase in weight gain decreased over the aging time, as expected, suggesting that the infusion of free water within the pores was reaching saturation.

### 3.3. Thermal Properties (TGA and DSC)

**Figure 8:**
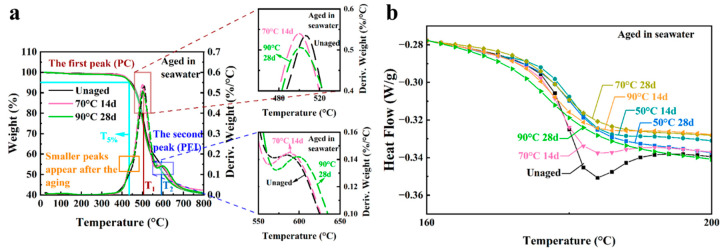
Thermal properties for unaged and aged 3D-printed Ultem 9085 samples in seawater. (**a**) TGA and D-TGA curves; (**b**) DSC curves.

The thermal degradation behavior of the 3D-printed Ultem 9085 is shown in Figure 8a for the unaged and post-aging conditions. The D-TGA curve for the unaged samples shows two peaks, which are evident at 506 °C and 594 °C. According to Padovano et al., thermal degradation of Ultem 9085 in a nitrogen environment involved two components, which included the degradation of PC at a lower temperature and PEI at a higher temperature [21]; indeed, that two stage response is noted in the D-TGA curves here. 

To quantify the degradation of the 3D-printed Ultem 9085 materials due to seawater exposure, the initial decomposition temperature and the temperature for the two maximum degradation peaks were determined from the TGA responses. The initial decomposition temperature here is defined as the temperature with 5% weight loss (T5%); the temperature for the first highest degradation peak (PC) and the second highest degradation peak (PEI) are defined as T1 and T2, respectively. These three values (T5%, T1 and T2) are reported for the Ultem 9085 samples in Table 4 for the experimental aging conditions. The T5% and T1 values for the treated specimens were all lower than the value for the untreated specimens, whereas the T2 values increased after being treated in the marine environment. Also evident in Figure 8a, another small peak appears near 440 °C after the samples underwent accelerated aging.

Relaxation enthalpy (*δ*_H_) was obtained by calculating the enclosed areas between the DSC trace and the baseline fitted through the DSC trace after the glass transition temperatures [49]. The DSC curves for the virgin and aged Ultem 9085 samples are shown in Figure 8b and Figure S2a. Tg, relaxation enthalpy and peak temperature (Tp) before and after seawater exposure are listed in Table 4. It has been widely stated that the Tg for Ultem with over 10% PC blended with PEI will lead to phase separation that is evident in the material [50,51,52,53]. As a result, there are often two distinct Tgs observed in the PEI/PC blend product, which are produced by the PC-rich and PEI-rich regions. However, only one Tg was observed in this study for Ultem 9085 at 180.3 °C. That agrees with the Tg reported in a previous work for an Ultem 9085 filament, where the DSC curve exhibited a single peak and Tg of approximately 180 °C [43]. Apart from the samples exposed at 50 °C for 14 days, Tg decreased after aging in seawater, particularly with increasing exposure temperatures. Also evident in Figure 8b, there was an endothermic enthalpy relaxation peak in the signature for the untreated sample and, after accelerated aging, the treated samples had a lower relaxation enthalpy for all the conditions.

### 3.4. Analysis of Molecular Composition (Raman)

**Figure 9:**
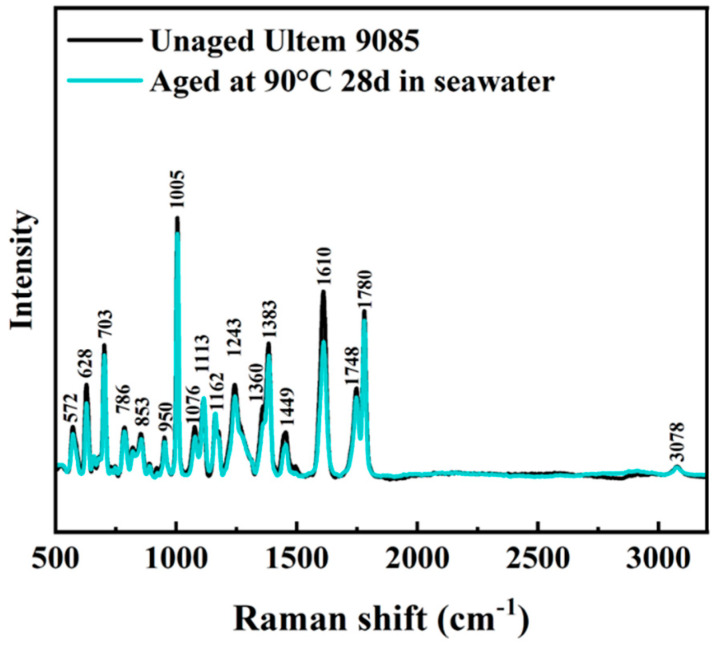
Raman spectra for unaged and aged at 90 °C for 28 days Ultem 9085 material in seawater.

Selected experimental Raman spectra for t Ultem 9085 in the unaged condition and aged at 90 °C for 28 days are shown in Figure 9, ranging from 500 to 3200 cm^−1^. The spectral properties of Ultem 9085 relies on the two major polymer components, i.e., PEI and PC [54]. According to a comparison of the experimental spectra with the WiRE software (Version 5.2) reference library and the Raman spectra of PC and Ultem 1000 (Figure S3), there was no new peak caused by exposure of Ultem 9085 to accelerated aging in seawater. The Raman spectra for Ultem 9085 are shown in Figure S4a of the SI for samples aged at the different conditions. No new peaks in the spectra were produced after accelerating aging in the artificial seawater, regardless of the aging condition. However, there was some variation in the intensity of different peaks across the aging conditions, which suggests potential differences in the degree of hydrolysis or physical aging reactions.

## 4. Discussion

Results of microscopy and µCT imaging showed that the microstructure of the printed samples exhibited a substantial degree of porosity regardless of the infill pattern (Figure 7a). Considering marine applications, pores within the microstructure are detrimental for at least two reasons, including the potential for premature failures due to the stress concentrations and water ingestion within the pores that facilitates changes in weight and a greater extent of water-related degradation. Previous studies conducted on injection-molded PEI and injection-molded PC reported that the water absorption reached an equilibrium of approximately 0.5 wt% and 0.6 wt% after approximately 4 days [55,56]. Other studies conducted with Ultem 9085 specimens produced by FFF and immersed in a high-pressure saltwater environment and real marine environment at around 15 °C reported that the increase in weight saturated at 9 wt% after 8 days and at 4.5% after 6 months [15,34]. In the present study, the maximum increase in wt% for the Ultem 9085 samples exceeded 7%, which falls within the rather wide reported range. Although the rate of water uptake decreased after 14 days exposure, it was not clear that an equilibrium was reached after 28 days (Figure 7b). Clearly, the water uptake of the Ultem 9085 material exceeded that of both the injection-molded PEI and PC, as expected, and the primary factor was the printed microstructure. Based on the visible porosity, the degree of weight increase was expected to depend on the printing parameters and part geometry, as both could contribute to the extent of porosity in the 3D-printed components. That could also cause differences in the degree of water uptake between benchtop studies with coupons and more completed printed components, an issue of practical importance.

### 4.1. Property Changes Following Accelerated Aging 

The thermal stability of polymers depends on the molecular weight, degree of crosslinking and crystallinity [57]. As such, not all polymers undergo the same degree of aging-related degradation. Hydrothermal aging reportedly does have a significant effect on the properties of PC and is associated with chain scission [58,59,60], and hydrolytic attack of this polymer reportedly happens more easily in a high pH and high-temperature environment. Indeed, the decrease in T5% and T1 peak values and appearance of the new peak in Figure 8a can be attributed to scission of the PC chains caused by hydrolysis reaction in the artificial seawater. In contrast, the thermal stability of PEI could be improved after prolonged exposure to seawater at elevated temperatures. Crosslinking of PEI occurs above 320 °C, which is much higher than the aging temperature performed here [61]. However, hydrogen bonds can develop that enhance the thermal stability of polymers in general [57]. In PEI, the increased thermal stability can result from the formation of hydrogen bonds, as well as due to changes in the crystallinity or structure of PEI in marine environments at an elevated temperature [62]. 

The Tg of polymers depends on the polymer chain mobility. The relaxation enthalpy is frequently used to evaluate physical aging of polymers. In the Ultem 9085 samples subjected to accelerated aging, there were four contributions to the polymer chain mobility, which included chain scission by hydrolysis to PC, the formation of interchain hydrogen bonding in PEI, the plasticization posed by water molecules and the physical aging in both PC and PEI. The presence of a relaxation enthalpy peak in the unaged samples revealed that there was already some thermal history on the sample. The process of physical aging involves a competition between aging and rejuvenation [49]. It has been reported that the earlier more dominant rejuvenation process can produce an increase in Tg and Tp and a reduction in *δ*_H_ [49]. The increase in Tp and the decrease in *δ*_H_ occurring in the aged samples suggested a more significant rejuvenation. For the samples aged at 50 °C for 14 days, the incremental increase in Tg appeared to result from the more severe physical aging driven by the elevation in temperature in comparison with the contribution of water plasticization due to the limited time that evolved for diffusion. With longer exposure periods and higher temperatures, which drive a corresponding increase in water diffusion, the plasticization effect of water and the chain scission of PC can lead to the decrease in Tg, as evident for Ultem 9085 aged at 90 °C for 28 days.

Regarding the Raman spectra in Figure 9, no new peaks were evident after exposure of Ultem 9085 to seawater degradation. Physical aging at an elevated temperature can decrease the free volume, which can lead to changes in infrared spectra intensity [63]. The C-H bonds in both polymers generate peaks around 3000 cm^−1^ [54]. The intensity changes in C=O stretching at 1748 cm^−1^ and 1780 cm^−1^ are reflective of the hydrolysis occurring to PC and the influence of hydrogen bonding in PEI and/or the changes in the molecular structure of PEI [64,65]. To further confirm the development of hydrogen bonds in the system, a wavelength over 3200 cm^−1^ is needed [62], which is of interest but left for future work. 

While the changes in thermal properties of Ultem 9085 showed some degree of degradation after accelerating aging in seawater, there was an unexpected improvement in the mechanical properties and the potential mechanisms were not evident in the Raman spectra. As a result, accelerated aging of another group of Ultem 9085 samples was performed in an air environment (sans seawater). This effort was performed using the Type 4 configuration with −45/45° and 0/90° Ultem 9085 sample groups. These two groups were chosen and did not include the Type 1 specimens due to the larger number of Type 1 specimen failures in the out-of-gauge section, as shown in Figure 4. The purpose was to investigate the influence of elevated temperatures on the material independent of the hydrolysis driven by seawater exposure. Akin to the evaluation after seawater aging, tensile tests, TGA, DSC and Raman spectroscopy were performed, and the results were compared to those from seawater aging.

### 4.2. Comparisons of Air vs. Marine Aging 

**Figure 10:**
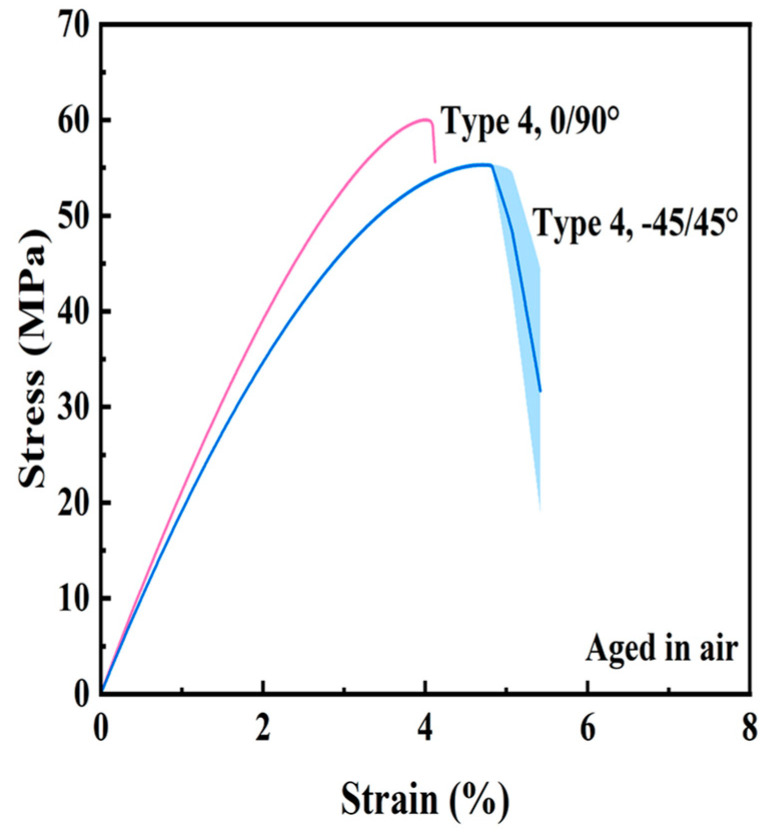
Stress–strain response of Ultem 9085 aged in air at 90 °C for 28 days.

**Figure 11:**
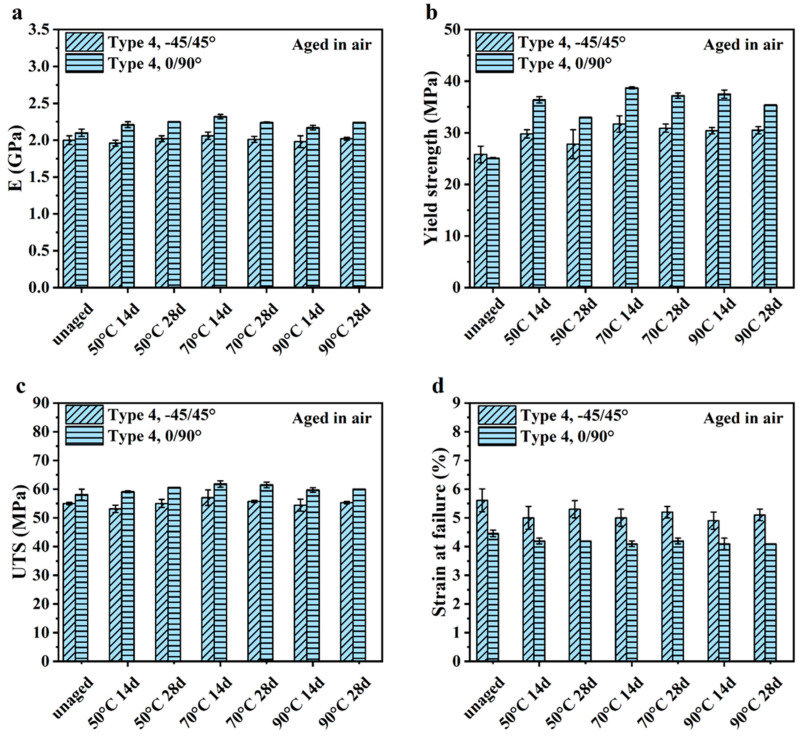
Mechanical properties of different types of unaged Ultem 9085 and aged Ultem 9085 calculated from their strain–stress curves in air. (**a**) Elastic modulus (E); (**b**) yield strength; (**c**) ultimate tensile strength (UTS); (**d**) strain at failure.

Stress–strain curves for the Type 4 Ultem 9085 samples after accelerated aging in air at 90 °C for 28 days are shown in Figure 10. The mechanical properties were determined after air aging and are summarized in Figure 11; the complete data set is presented in the Supplemental Information (SI, Tables S6–S9). Akin to results from the seawater aging, some of the specimens failed prematurely in the out-of-gauge section and the tensile data for those failed specimens were excluded when estimating the mechanical properties. All results from the statistical comparisons are detailed in SI Tables S6–S9. 

Figure 11a presents the elastic modulus of the specimens. As evident from these responses, the E of the Type 4 0/90° specimens increased after aging in air, which was consistent with results of the Type 4 0/90° specimens after aging in seawater. Although there was a very small increase in E of the Type 4 −45/45° specimens after aging at 70 °C for 14 days, there was no significant increase in E for the remainder of the aging conditions. That contrasted results after aging in seawater, where the increase in E was significant for all conditions and temperatures. Results for the yield strength of Ultem 9085 exposed to accelerated aging in air are presented in Figure 11b. Accelerated aging in air caused an increase in the yield strength, which was consistent with seawater aging. The yield strength of both sample groups that underwent air aging exhibited a significant increase, except for the Type 4 0/90° samples aged at 50 °C for 28 days and 90 °C for 28 days and Type 4 −45/45° aged at 50 °C for 28 days. When comparing the increase in yield strength for air and seawater (Figure 7b), air aging caused a higher increase in yield strength of the Type 4 0/90° samples in all conditions, excluding at 90 °C for 28 days. In contrast, the seawater aging led to a higher yield strength of the Type 4 −45/45° samples in all the conditions, excluding at 90 °C for 14 days.

Results for UTS in Figure 11c show that accelerated aging of the Type 4 0/90° specimens caused some moderate increases in UTS, whereas there were no marked changes in the UTS of −45/45° specimens after accelerated air aging. Recall that after exposure to seawater, the UTS of both Type 4 sample configurations increased, especially at the highest temperature. In contrast to aging in seawater, in air, the highest temperature (90 °C) did not result in a significant difference in UTS between the control samples and the aged samples. Rather the UTS of the Type 4 −45/45° samples exposed to air was significantly different from the UTS of the virgin samples at 50 °C for 14 days and at 70 °C for 28 days. 

Regarding the strain to failure in Figure 11d, accelerating aging in air caused a reduction in strain to failure regardless of the printing infill orientation, which was consistent with the responses in seawater. In air, a significant decrease in strain at failures was observed for Type 4 0/90° samples aged at 70 °C for 14 days and Type 4 −45/45° samples aged at all temperatures for 14 days. In contrast, in the seawater environment test, the significant decrease in elongation failure of the Type 4 0/90° samples and Type 4 −45/45° samples only occurred at the highest temperature, i.e., at 90 °C for 14 days and for 28 days. 

Measures of the COV for the samples aged in air are listed in Table S10. When comparing the responses from the two different aging environments, the specimens aged in seawater at elevated temperatures exhibited a higher variation in the E. Regarding yield strength, the Type 4 0/90° samples exposed to seawater had a larger COV at a higher temperature than the samples exposed to air. Regarding yield strength, the largest COV for the Type 4 −45/45° samples appeared from air aging at a lower temperature, whereas the largest COV for seawater aging happened at the highest temperature. Interestingly, for the Type 4 0/90° samples, the largest COV in UTS occurred for the control condition rather than after aging in either environment, undoubtedly a function of the microstructure. Regarding UTS, the largest COV for the −45/45° samples resulted from aging in seawater compared with air aging. Regarding the Type 4 −45/45° samples, they underwent large variations in elongation failure in both aging environments. In contrast, the largest aging-related variation in the strain at failure of the Type 4 0/90° samples occurred in the marine condition.

**Figure 12:**
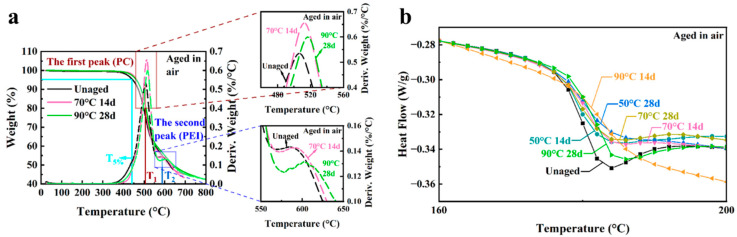
Thermal properties for unaged and aged 3D-printed Ultem 9085 samples in air. (**a**) TGA and D-TGA curves; (**b**) DSC curves.

The TGA and D-TGA curves of Ultem 9085 after air aging is shown in Figure 12a. Results from the spectra for the parameters used to quantify the thermal degradation (T5%, T1 and T2) are summarized in Table 5. Distinct from the material treated in seawater, no smaller peaks at around 440 °C were produced by aging in air, which suggests that hydrolytic-related degradation in the PC did not occur. In addition, the T5%, T1 and T2 all increased after exposure to air at elevated temperatures.

The DSC curves for the 3D-printed samples are presented in Figure 12b and Figure S2b for the unaged and air-aged conditions. The samples treated in air at 70 °C for the longer duration exhibited an endothermic melting peak at around 220 °C, which suggests that crystallization occurs during air aging. Tg values of the samples aged in air are listed in Table 5 and reflect that there were some small changes, which were related to the rejuvenation and physical aging process. Yet, the variation in Tg from air aging is not as much as that for seawater. At 50 °C, it was obvious that the *δ*_H_ and Tp values decreased faster in seawater than those properties in air aging. However, at the higher temperatures (70 °C and 90 °C) in air, the decrease and increase in *δ*_H_ and Tp, respectively, and shorter time to reach a zero *δ*_H_, revealed an increase in the rate of physical aging. 

**Figure 13:**
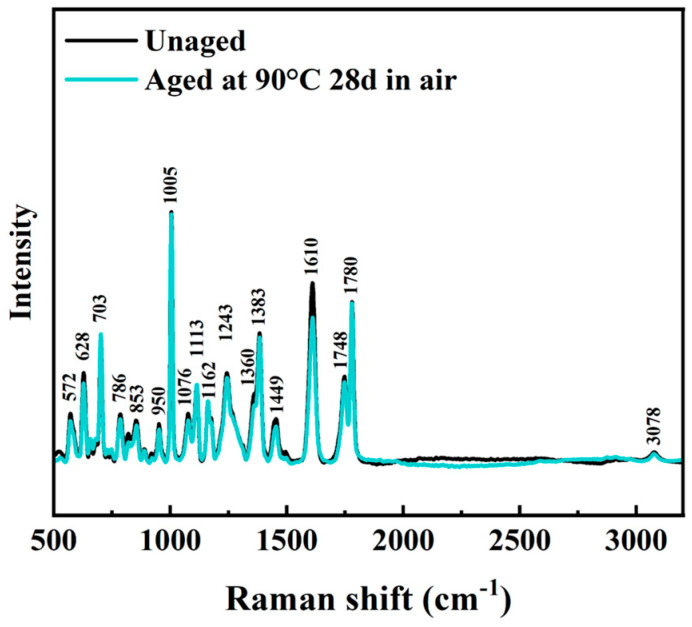
Raman spectra for unaged and aged at 90 °C for 28 days Ultem 9085 material in air.

A comparison of the Raman spectra for the unaged printed sample and for a representative sample aged in air (90 °C for 28 days) is shown in Figure 13. The Raman spectra for the Ultem 9085 samples at all air-aging conditions are shown in Figure S4b. Identical to Ultem 9085 aged in seawater, no new peaks were identified in the spectra after accelerating aging in air. While there were some changes in the intensity at different peaks that were apparent after air aging, they were not as distinct as those in the marine aging. Indeed, this was expected and confirmatory of the additional contributing mechanisms active in the seawater aging condition, including water plasticization, hydrolytic reactions and physical aging. Only some degree of crystallization and physical aging occurred in air. By comparing the thermal property changes of the Ultem 9085 material after the two aging conditions, it became apparent that hydrolysis and plasticization in the seawater environment had a more pronounced effect at 90 °C, which could also be observed in the Raman spectra.

Accelerated aging in seawater had the most effect on the E and yield strength of Ultem 9085 and was most evident from the responses for the Type 4 samples in Figure 3a,b. Both properties increased with aging time and temperature. Yet, for aging in air, only the yield strength of the Type 4 samples exhibited substantial changes, as noted in Figure 11b; the increase in strength was significant and reached nearly 40%. Through a comparison of the changes in mechanical properties for air and seawater, it can be noticed that E was more affected by accelerating aging in seawater. In addition, the mechanical properties of both Type 4 sample configurations varied differently with the increase in aging period. Nevertheless, overall, the yield strength exhibited the most change after exposure to aging in both conditions. Mechanistically, the increase in strength was associated with the microstructural effects on the initiation of chain sliding. It was hypothesized that chain sliding was resisted by the increase in crystallinity and physical aging. The influence of hydrolytic mechanisms in seawater resulted in a more complex concert of mechanisms. 

### 4.3. Relevant Prior Work

Reported studies on the durability of Ultem 9085 after exposure to marine environments are limited. Padovano et al. [32] evaluated the aging behavior of 3D-printed Ultem 9085 after dry storage at 100 °C, cyclic temperature change from −40 to 90 °C in a humid condition and thermal shock from 70 to −20 °C. They used three-point flexural tests and quantified the E and flexural strength of the aged samples. Interestingly, the samples that underwent gradual thermal cycling in a humid environment underwent a significant increase in E and flexural strength [32]. However, thermal shock caused a decrease in E and strength [32]. Alternatively, Bagsik et al. [21] prepared Ultem 9085 coupons cut from panels printed by FFF and submerged them into real seawater at a low temperature (approximately 15 °C) for 6 months to investigate the changes in mechanical behavior. Results showed that aging in seawater caused an increase in E, yield strength and UTS, as well as a reduction in the strain at failure [21]. These results are largely consistent with those of the present study. However, the contributing mechanisms responsible for the mechanical property variations were not evaluated in these investigations [21,32]. Understanding the mechanisms involved appears critical for distinguishing the applicability of the results to a design environment. 

There has been some relevant work performed on changes to the durability of other polymers printed by FFF and then subjected to moist and/or submerged environments. In general, these studies have reported that there is some degree of degradation in the mechanical properties. For instance, Chaudhary et al. immersed a variety of polymers (ABS, PLA, PETG, etc.) printed by FFF in unspecified seawater at temperatures ranging from 30 to 45 °C [66]. After roughly one month of exposure, the changes in E and water diffusivity were evaluated [66]. They found that the 3D-printed polymers had a higher water diffusivity than their traditionally manufactured polymer counterpart [66]. Interestingly, the E of the 3D-printed material exhibited some degradation after the 4-week period, which was assumed to be caused by contributions from the water to the secondary bonds in the respective polymers [66]. El Magri et al. studied the influence of physical aging on the mechanical properties of a 3D-printed blend of PEEK/PEI in dry (120 °C) and 85% relative humidity (70 °C) environments after 42 days of aging [67]. Based on the results of tensile testing, the E of the material decreased considerably in the humid environment, whereas UTS did not appear affected by either the dry or moist aging conditions [67]. They attributed the degradation to plasticization of the polymer chains caused by the water and internal stresses caused by the voids between deposited layers. 

Ho et al. performed physical aging experiments on polycarbonate specimens produced by traditional manufacturing methods at temperatures from 60 to 120 °C and for almost 15 days; changes in structure were evaluated with DSC [49]. They found that conformational changes in the polymer chains driven by physical aging led to a more parallel aligned chain distribution, which would foster an increase in crystallinity and improvements in stiffness and yield strength [49,68,69,70]. In the present accelerating aging study, the significant increase in yield strength could be explained by the contributions of physical aging that was accelerated at elevated temperatures. With respect to the higher E after the seawater aging, water could increase the mobility of the chains, which could contribute to the more significant increase in E within seawater at elevated temperatures [68]. A longer period of storage would likely enable the effects of hydrolytic degradation to become more prominent. As such, accelerated aging studies performed to evaluate the durability of polymers processed by FFF is a useful approach to understand mechanistic contributions to property changes with time. However, the results may not be representative of those that are active in the application environment due to the competing mechanisms and rates of degradation. Although one of the goals of this effort was to develop a model for the aging response, the plurality in active mechanisms, the short aging period and the issues with out-of-gauge failures severely limited the opportunities for modeling. Nevertheless, the investigation was not a failure. Overall, the results demonstrate that degradation of Ultem 9085 can be expected under exposure to marine environments and that it manifests through a reduction in the strain to failure, which results in embrittlement. It must be considered for applications that will involve exposure to marine environments. Furthermore, making adjustments to the design to accommodate this degradation is not sufficient and the degree of degradation is expected to increase over time.

### 4.4. Limitations 

The findings from this investigation showed that there were significant changes in the mechanical properties of Ultem 9085 with accelerated aging and that there were several contributing mechanisms to the degradation. Interestingly, the relative effects of aging were dependent on the sample type (ASTM Type 1 or 4) and infill pattern, which was related to the porosity, differences in water penetration and their importance to the structural behavior. While the findings help to advance our understanding of aging-related degradation in Ultem 9085, there were limitations to this study that should be considered. One obvious concern regarding the aging condition was that a simple laboratory marine environment was used. When applying the material in a real marine environment, there are many additional threats of potential relevance, including UV light, biofouling from marine organisms, different salinity and ions and potential hydrostatic pressure, which could simultaneously and synergistically contribute to deterioration. In addition, while there were some significant changes in mechanical behavior, the extent of overall degradation in strength or strain to failure was rather limited. Admittedly, the accelerated aging protocol involved a limited time, with a maximum of 28 days; more substantial degradation could arise with a longer aging period. Another concern in this study was the specimen type and number of unacceptable failures. It is becoming widely recognized that the ASTM standard specimens for characterizing the tensile properties of polymers are not reliable for FFF [28,34,46,47,48,71]. Premature failures occurred due to the voids between the infill and contour outlines and the inherent stress concentration produced by the sample design. For instance, results of tension testing for the Type 1 −45/45° samples were not valid. In addition, there was some loss in the data for some samples with Type 1 0/90°, Type 4 0/90° and Type 4 −45/45° configurations due to invalid failure locations. Based on these results, further work should be performed to find the most reliable specimen geometry to be enrolled for future aging evaluations. In addition, longer time periods of aging should be utilized to examine whether the degradation continues to evolve with further time or whether it reaches an equilibrium state. 

## 5. Conclusions

An experimental investigation was performed on 3D-printed Ultem 9085 produced by FFF to characterize the resistance to degradation under accelerated aging involving exposure to seawater. Based on the results and analyses performed, the following conclusions were drawn:

1. Results of accelerated aging in seawater showed there was a significant increase in elastic modulus and both yield and ultimate tensile strength. However, there was a decrease in strain to failure. In air, there was also an increase in yield and ultimate tensile strength. The most substantial change in properties with aging was in the magnitude of yield strength with increasing temperature.
2. Results from TGA revealed that the accelerated aging protocol caused hydrolytic degradation in the PC of Ultem 9085 and appeared most responsible for the decrease in strain to failure. The degradation in strain to failure reached significance when the material was aged in seawater at the highest temperature (90 °C).
3. According to the results of Raman analysis, physical aging occurred in PEI and PC and contributed to the improvement in elastic modulus and strength, with accelerated aging. The process of accelerated aging in air resulted in physical aging without hydrolytic degradation and showed that there was negligible degradation in strain to failure with increasing aging time or temperature.
4. Premature or unacceptable failures occurred in some of the tensile specimens of both types (Type 1 and Type 4) due to the voids located between the infill and contour outlines of the tensile specimens. These internal defects posed inherent stress concentrations and were most detrimental to the Type I tensile specimens. There appears to be a need to modify the specimen geometry of the ASTM standard D638 to support more reliable characterization of the mechanical properties of polymers if they are produced by FFF.
5. When conducting accelerating aging tests of polymers in marine environments, it is important to recognize that physical aging is one of the contributing mechanisms. Consequently, choosing a proper aging protocol that excludes the effects of physical aging may be necessary to isolate the effects of the other aging mechanisms.

## Figures and Tables

**Table 1:** Aging plan for Ultem 9085 in substitute sea water and air.

Temperature (°C)	Aging Period (Days)	Samples in Seawater	Samples in Air
50	14	Type 4, −45/45°, Type 4, 0/90°, Type 1, 0/90°	Type 4, −45/45°, Type 4, 0/90°
50	28	Type 4, −45/45°, Type 4, 0/90°, Type 1, 0/90°	Type 4, −45/45°, Type 4, 0/90°
70	14	Type 4, −45/45°, Type 4, 0/90°	Type 4, −45/45°, Type 4, 0/90°
70	28	Type 4, −45/45°, Type 4, 0/90°	Type 4, −45/45°, Type 4, 0/90°
90	14	Type 4, −45/45°, Type 4, 0/90°, Type 1, 0/90°	Type 4, −45/45°, Type 4, 0/90°
90	28	Type 4, −45/45°, Type 4, 0/90°, Type 1, 0/90°	Type 4, −45/45°, Type 4, 0/90°

**Table 2:** Roughness of the Ultem 9085 samples produced by FFF.

Samples	Direction 1		Direction 2		Edge	
	Ra (μm)	Rz (μm)	Ra (μm)	Rz (μm)	Ra (μm)	Rz (μm)
Type 1, 0/90°	11	48	10	48	2	11
Type 1, −45/45°	19	102	23	119	2	9
Type 4, 0/90°	12	62	11	66	2	11
Type 4, −45/45°	32	175	33	173	1	7

**Table 3:** Areal porosity in Type 4 Ultem 9085 samples and potential water uptake range.

Orientation	Areal Porosity (%)	Potential Water Uptake Range (%)
−45/45°	7.34 ± 1.69	7.21 ± 1.66
−45/45°	8.11 ± 1.50	7.98 ± 1.48
0/90°	8.43 ± 5.05	8.29 ± 4.97
0/90°	10.50 ± 5.19	10.33 ± 5.10

**Table 4:** Thermal properties of unaged and aged 3D-printed Ultem 9085 samples before and after seawater exposure. Note that “d” indicates days.

Samples	T5% (°C)	T1 (°C)	T2 (°C)	Tg (°C)	*δ*_H_ (J/g)	Tp(°C)
Unaged	444.7	506.4	584.7	180.3	0.148	184
50 °C 14 d	443.2	501.9	589.1	180.7	0.01	188
50 °C 28 d	444.2	502.3	595.9	179.8	0.003	190
70 °C 14 d	441.1	499.9	588	179.1	0.042	184
70 °C 28 d	440.9	502.5	593.2	179.1	0.029	190
90 °C 14 d	435.3	498.8	590.1	178.1	0.028	186
90 °C 28 d	438.6	501.3	597.5	176.8	0	

**Table 5:** Thermal properties of unaged and aged Type 4 3D-printed Ultem 9085 samples before and after air exposure. Note that “d” indicates days of exposure.

Samples	T5% (°C)	T1 (°C)	T2 (°C)	Tg (°C)	*δ*_H_ (J/g)	Tp(°C)
Unaged	444.7	506.4	584.7	180.3	0.148	184
50 °C 14 d	462.2	512	602	179	0.121	184
50 °C 28 d	459	516	598	179.8	0.016	186
70 °C 14 d	464.4	512	592	181.7	0.012	186
70 °C 28 d	464.7	510	598	179.8	0.052	184
90 °C 14 d	464.5	516	610	182.8	0	
90 °C 28 d	462.7	516	602	179.3	0.102	186

## Data Availability

Data is available upon request.

## Supplementary Materials

The following supporting information can be downloaded at: https://www.mdpi.com/article/10.3390/polym16030350/s1.
