# Supplementary material for: Durability of Ultem 9085 in Marine Environments: A Consideration in Fused Filament Fabrication of Structural Components
Source: Polymers (Basel). 2024 Jan 28;16(3):350. doi: 10.3390/polym16030350 (PMC10856876; doi:10.3390/polym16030350)
Supplement: Supplementary file 1 [file polymers-16-00350-s001.zip › polymers-2784767-supplementary.pdf]

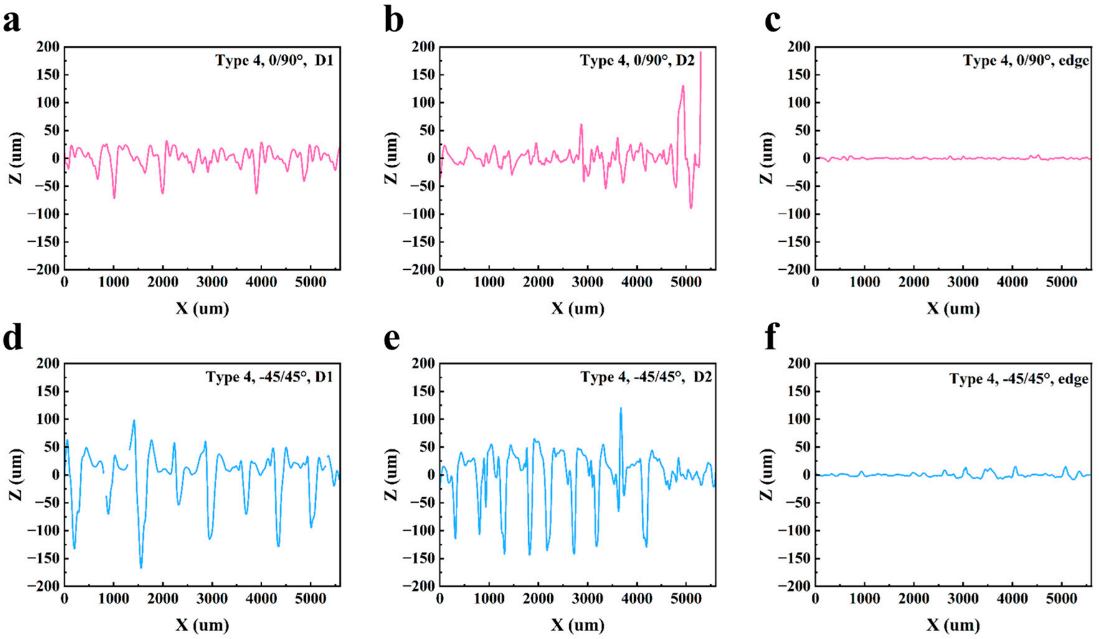

Figure S1: Roughness profile of the unaged Type 4 Ultem 9085 samples. a) 0/90° orientation, D1; b) 0/90° orientation, D2; c) 0/90° orientation, edge; d) -45/45° orientation, D1; e) -45/45° orientation, D2; f) -45/45° orientation, edge.

Table S1: t-test result for the E of the Ultem 9085 samples after different exposures in seawater. Type 1, 0/90° specimens, Type 4, 0/90° specimens and Type 4, -45/45° specimens are replaced by a, b and c in the table. The significantly different data is highlighted in yellow.

Mechanical property	Environment	Condition	Samples	Mean	Standard deviation	Observations	Variance	Degree of freedom	t Stat	P(T<=t) two tail	t critical two-tail
E (GPa)	Seawater	Unaged	a	2.2	0.04	3	0.002				
			b	2.1	0.05	3	0.002				
			c	2	0.06	4	0.003				
		50°C 14 d	a	2.3	0.05	5	0.002	5	2.06	0.09	2.57
			b	2.3	0.05	2	0.002	2	4.5	<0.05	4.30
			c	2.1	0.09	5	0.007	7	3.01	<0.02	2.36
		50°C 28 d	b	2.3	0.04	3	0.002	4	4.7	<0.01	2.78
			c	2.2	0.13	5	0.02	6	3.6	<0.02	2.45
		70°C 14 d	b	2.3	0.04	4	0.001	4	10.82	<0.001	2.78
			c	2.3	0.08	5	0.007	7	6.33	<0.01	2.36
		70°C 28 d	b	2.2	0.002	2	4.00E-06	2	4.68	<0.05	4.30
			c	2.2	0.11	5	0.01	6	4.41	<0.01	2.45
		90°C 14d	a	2.3	0.03	3	0.001	4	2.91	<0.05	2.78
			b	2.5	0.04	3	0.002	4	10.9	<0.01	2.78
			c	2.3	0.13	5	0.02	6	5.26	<0.01	2.45
		90°C 28 d	a	2.3	0.02	3	4.00E-04	3	3.26	<0.05	3.18
			b	2.5	0.10	3	0.01	3	6.87	<0.01	3.18
			c	2.2	0.08	5	0.006	7	5.51	<0.01	2.36

Table S2: t-test result for the yield strength of the Ultem 9085 samples after different exposures in seawater. Type 1, 0/90° specimens, Type 4, 0/90° specimens and Type 4, -45/45° specimens are replaced by a, b and c in the table. The significantly different data is highlighted in yellow.

Mechanical property	Environment	Condition	Samples	Mean	Standard deviation	Observations	Variance	Degree of freedom	t Stat	P(T<=t) two tail	t critical two-tail
Yield strength (MPa)	Seawater	Unaged	a	23.9	2.1	3	4.45				
			b	25.1	0.1	3	0.01				
			c	25.8	1.6	4	2.51				
		50°C 14 d	a	28.0	1.6	5	2.47	3	2.89	0.06	3.18
			b	34.7	0.2	2	0.03	2	72.28	<0.01	4.30
			c	31.6	0.8	5	0.64	4	6.74	<0.01	2.78
		50°C 28 d	b	30.9	1.1	3	1.11	2	9.54	<0.02	4.30
			c	30.1	0.9	5	0.79	4	4.90	<0.01	2.78
		70°C 14 d	b	36.9	1.4	4	2.07	3	16.34	<0.01	3.18
			c	32.3	1.0	5	1.05	5	7.10	<0.01	2.57
		70°C 28 d	b	33.1	0.8	2	0.57	1	14.88	<0.05	12.71
			c	31.2	0.9	5	0.77	4	6.13	<0.01	2.78
		90°C 14 d	a	25.9	1.4	3	2.06	4	1.31	0.26	2.78
			b	33.8	1.4	3	1.85	2	11.11	<0.01	4.30
			c	30.1	4.1	5	16.60	5	2.18	0.08	2.57
		90°C 28 d	a	25.0	1.9	3	3.74	4	0.63	0.56	2.78
			b	36.7	2.8	3	8.07	2	7.11	<0.02	4.30
			c	34.1	2.6	5	6.91	7	5.87	<0.01	2.36

Table S3: t-test result for the UTS of the Ultem 9085 samples after different exposures in seawater. Type 1, 0/90° specimens, Type 4, 0/90° specimens and Type 4, -45/45° specimens are replaced by a, b and c in the table. The significantly different data is highlighted in yellow.

Mechanical property	Environment	Condition	Samples	Mean	Standard deviation	Observations	Variance	Degree of freedom	t Stat	P(T<=t) two tail	t critical two-tail
UTS (MPa)	Seawater	Unaged	a	51.3	1.5	3	2.19				
			b	58.1	1.9	3	3.52				
			c	55.0	0.4	4	0.17				
		50°C 14 d	a	50.2	1.2	5	1.49	4	-1.06	0.35	2.78
			b	58.6	1.4	2	0.20	2	0.40	0.73	4.30
			c	56.0	0.4	5	1.95	5	1.65	0.16	2.57
		50°C 28 d	b	58.3	0.5	3	0.28	2	0.15	0.90	4.30
			c	58.0	3.2	5	10.31	4	2.13	0.10	2.78
		70°C 14 d	b	60.8	0.9	4	0.77	3	2.28	0.11	3.18
			c	59.8	2.5	5	6.24	4	4.30	<0.02	2.78
		70°C 28 d	b	57.2	0.4	2	0.18	2	-0.83	0.49	4.30
			c	57.8	2.3	5	5.31	4	2.67	0.06	2.78
		90°C 14 d	a	50.0	3.4	3	0.44	3	-1.42	0.25	3.18
			b	60.0	1	3	0.90	3	1.51	0.23	3.18
			c	59.9	2.3	5	5.17	4	4.77	<0.01	2.78
		90°C 28 d	a	47.3	3.0	3	1.26	4	-3.76	<0.02	2.78
			b	62.7	1.1	3	1.24	3	3.65	<0.04	3.18
			c	57.5	1.4	5	1.91	5	3.85	<0.02	2.57

Table S4: t-test result for the strain at failure of the Ultem 9085 samples after different exposures in seawater. Type 1, 0/90° specimens, Type 4, 0/90° specimens and Type 4, -45/45° specimens are replaced by a, b and c in the table. The significantly different data is highlighted in yellow.

Mechanical property	Environment	Condition	Samples	Mean	Standard deviation	Observations	Variance	Degree of freedom	t Stat	P(T<=t) two tail	t critical two-tail
Strain at failure (%)	Seawater	Unaged	a	3.8	0.2	3	0.03				
			b	4.5	0.1	3	0.01				
			c	5.6	0.4	4	0.16				
		50°C 14 d	a	3.5	0.1	5	0.006	3	-2.86	0.06	3.18
			b	4.2	0.2	2	0.03	2	-1.56	0.26	4.30
			c	5.5	0.5	5	0.29	7	-0.37	0.73	2.36
		50°C 28 d	b	4.3	0.2	3	0.03	3	-1.12	0.34	3.18
			c	5.4	0.4	5	0.15	6	-0.70	0.51	2.45
		70°C 14 d	b	3.9	0.4	4	0.18	4	-2.62	0.06	2.78
			c	5.6	0.3	5	0.08	5	-0.11	0.92	2.57
		70°C 28 d	b	4.4	0.6	2	0.33	1	-0.04	0.98	12.71
			c	5.3	0.3	5	0.11	6	-1.11	0.31	2.45
		90°C 14d	a	3.4	0	3	0.001	2	-4.16	0.05	4.30
			b	3.5	0.1	3	0.01	4	-11.66	<0.01	2.78
			c	5.2	0.2	5	0.06	5	-1.90	0.12	2.57
		90°C 28 d	a	3.0	0.1	3	0.02	4	-6.58	<0.01	2.78
			b	3.9	0.3	3	0.08	3	-2.90	0.06	3.18
			c	4.8	0.2	5	0.04	4	-3.59	<0.03	2.78

Table S5: Coefficient of variation of the Ultem 9085 samples exposed to seawater. Type 1, 0/90°, Type 4, 0/90° specimens and Type 4, -45/45° specimens are replaced by a, b and c in the table. The highest COV in each column is highlighted in yellow.

**Mechanical properties**	**E (%)**			**Yield strength (%)**			**UTS (%)**			**Strain at failure (%)**		
Samples	a	b	c	a	b	c	a	b	c	a	b	c
unaged	1.9	2.4	2.9	8.8	0.4	6.1	3.0	3.2	0.7	4.3	2.5	7.0
50°C 14 d	2.1	2.0	4.1	5.6	0.5	2.5	2.4	0.8	2.5	2.2	3.8	9.7
50°C 28 d		2.8	5.8		3.4	2.9		0.9	5.5		3.9	7.0
70°C 14 d		1.5	3.7		3.9	3.2		1.5	4.2		11.1	5.1
70°C 28 d		0.1	5.1		2.3	2.8		0.7	4.0		12.9	6.1
90°C 14 d	1.3	1.6	5.5	5.6	4.0	13.5	1.3	1.6	3.8	0.8	2.3	4.7
90°C 28 d	0.9	3.9	3.6	7.7	7.7	7.7	2.4	1.8	2.4	4.3	7.3	4.3

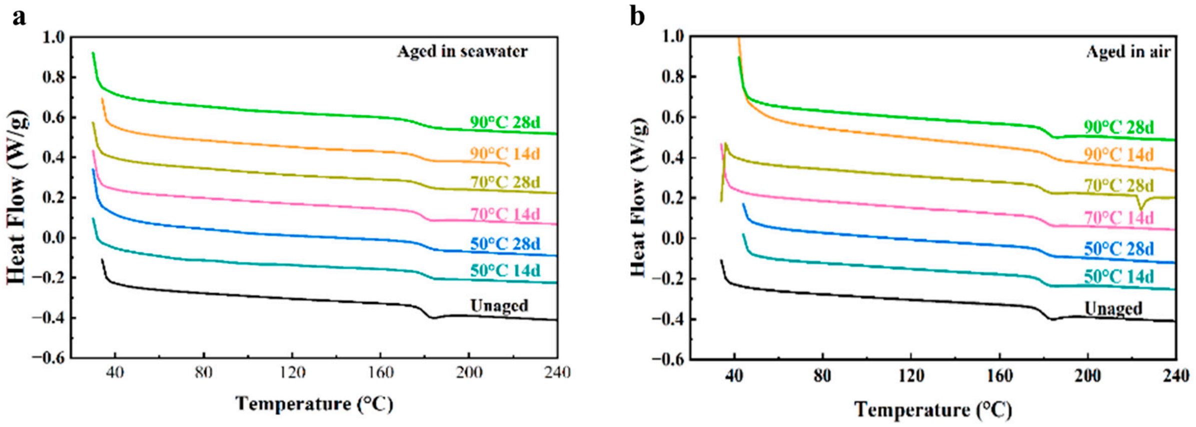

Figure S2: DSC curves for unaged and aged 3D-printed Ultem 9085 samples a) in seawater; b) in air.

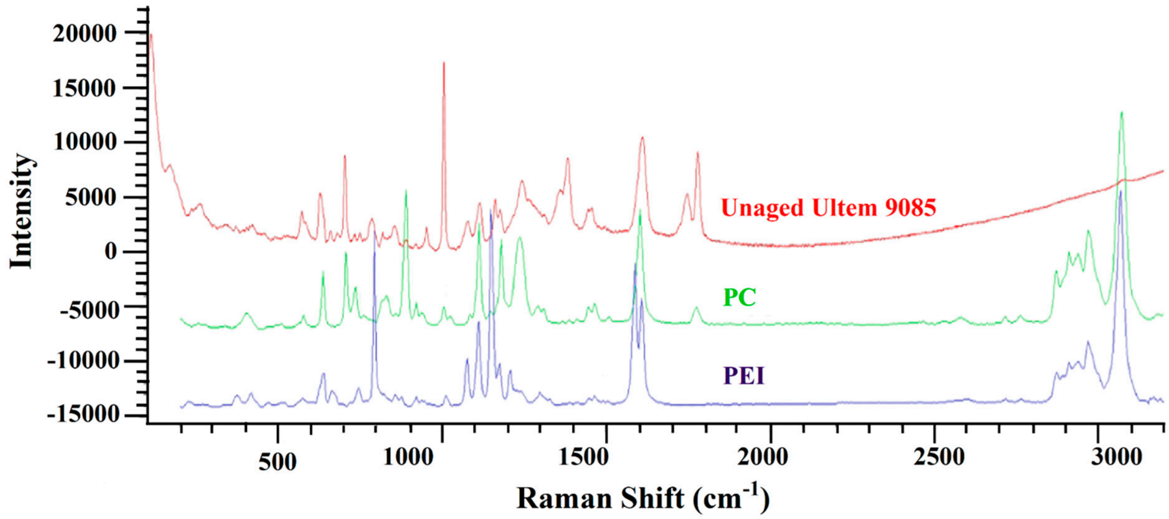

Figure S3: Raman spectra for unaged Ultem 9085, PC and PEI material from WiRE library.

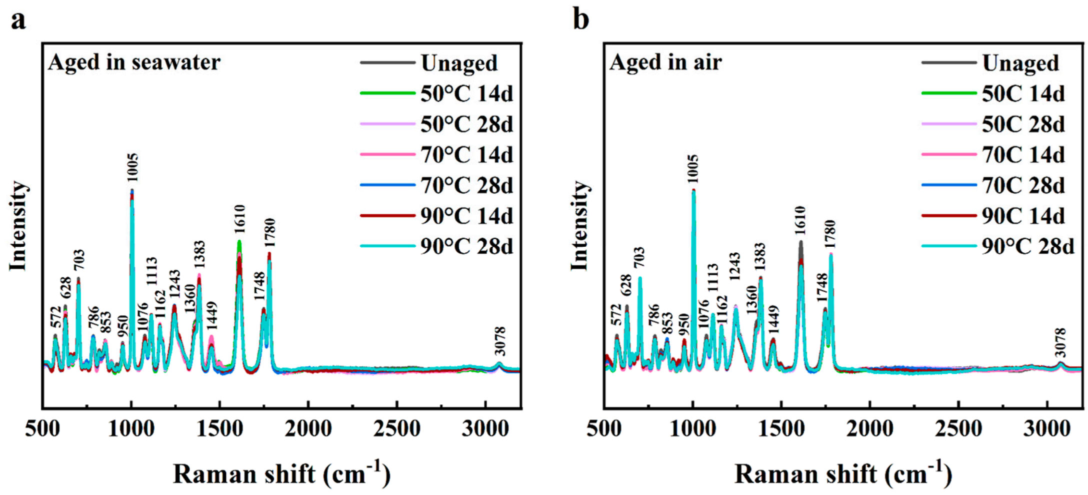

Figure S4: Raman spectra of Ultem 9085 after different treatment a) in seawater; b) in air.

Table S6: t-test result for the E of the Ultem 9085 samples after different exposures in air. Type 4, 0/90° specimens and Type 4, -45/45° specimens are replaced by b and c in the table. The significantly different data is highlighted in yellow.

Mechanical property	Environment	Condition	Samples	Mean	Standard deviation	Observations	Variance	Degree of freedom	t Stat	P(T<=t) two tail	t critical two-tail
E (GPa)	Air	Unaged	b	2.1	0.05	3	0.002				
			c	2.0	0.06	4	0.003				
		50°C 14 d	b	2.2	0.04	3	0.001	4	3.43	<0.03	2.78
			c	2.0	0.04	5	0.002	5	0.09	0.93	2.57
		50°C 28 d	b	2.2		1					
			c	2.0	0.04	4	0.002	6	1.63	0.15	2.45
		70°C 14 d	b	2.3	0.03	2	0.001	3	6.39	<0.01	3.18
			c	2.1	0.05	5	0.003	6	2.74	0.03	2.45
		70°C 28 d	b	2.2	0.01	3	7.2493E-05	2	5.16	<0.04	4.30
			c	2.0	0.04	5	0.001	5	1.46	0.20	2.57
		90°C 14 d	b	2.2	0.03	4	0.001	3	2.54	0.08	3.18
			c	2.0	0.08	5	0.006	7	0.51	0.63	2.36
		90°C 28 d	b	2.2		1					
			c	2.0	0.02	4	0.001	4	1.84	0.14	2.78

Table S7: t-test result for the yield strength of the Ultem 9085 samples after different exposures in air. Type 4, 0/90° specimens and Type 4, -45/45° specimens are replaced by b and c in the table. The significantly different data is highlighted in yellow.

Mechanical property	Environment	Condition	Samples	Mean	Standard deviation	Observations	Variance	Degree of freedom	t Stat	P(T<=t) two tail	t critical two-tail
Yield strength (MPa)	Air	Unaged	b	25.1	0.1	3	0.01				
			c	25.8	1.6	4	2.51				
		50°C 14 d	b	36.4	0.6	3	0.34	2	33.30	<0.01	4.30
			c	29.8	0.8	5	0.59	4	4.68	<0.01	2.78
		50°C 28 d	b	33.0		1					
			c	27.8	2.8	4	7.70	5	1.25	0.27	2.57
		70°C 14 d	b	38.7	0.2	2	0.03	1	98.37	<0.01	12.71
			c	31.7	1.6	5	2.55	7	5.54	<0.01	2.36
		70°C 28 d	b	37.2	0.5	3	0.24	2	41.85	<0.01	4.30
			c	30.9	0.8	5	0.64	4	5.86	<0.01	2.78
		90°C 14 d	b	37.5	0.8	4	0.62	3	31.21	<0.01	3.18
			c	30.4	0.6	5	0.34	4	5.48	<0.01	2.78
		90°C 28 d	b	35.4		1					
			c	30.5	0.7	4	0.43	4	5.54	<0.01	2.78

Table S8: t-test result for the UTS of the Ultem 9085 samples after different exposures in air. Type 4, 0/90° specimens and Type 4, -45/45° specimens are replaced by b and c in the table. The significantly different data is highlighted in yellow.

Mechanical property	Environment	Condition	Samples	Mean	Standard deviation	Observations	Variance	Degree of freedom	t Stat	P(T<=t) two tail	t critical two-tail
UTS (MPa)	Air	Unaged	b	58.1	1.9	3	3.52				
			c	55.0	0.4	4	0.17				
		50°C 14 d	b	59.1	0.3	3	0.07	2	0.92	0.45	4.3
			c	53.1	1.3	5	1.77	5	-2.90	0.03	2.57
		50°C 28 d	b	60.6		1					
			c	55.1	1.4	4	1.87	3	0.14	0.90	3.18
		70°C 14 d	b	61.8	1.1	2	1.13	3	2.74	0.07	3.18
			c	57.0	2.7	5	7.13	4	1.68	0.17	2.78
		70°C 28 d	b	61.5	1.0	3	0.97	3	2.75	0.07	3.18
			c	55.7	0.4	5	0.16	7	2.92	0.02	2.36
		90°C 14 d	b	59.7	0.8	4	0.57	2	1.37	0.31	4.30
			c	54.4	2.1	5	4.26	4	-0.54	0.62	2.78
		90°C 28 d	b	60.0		1					
			c	55.3	0.4	4	0.14	6	-1.36	0.22	2.45

Table S9: t-test result for the strain at failure of the Ultem 9085 samples after different exposures in air. Type 4, 0/90° specimens and Type 4, -45/45° specimens are replaced by b and c in the table. The significantly different data is highlighted in yellow.

Mechanical property	Environment	Condition	Samples	Mean	Standard deviation	Observations	Variance	Degree of freedom	t Stat	P(T<=t) two tail	t critical two-tail
Strain at failure (%)	Air	Unaged	b	4.5	0.1	3	0.01				
			c	5.6	0.4	4	0.16				
		50°C 14 d	b	4.2	0.1	3	0.02	4	-2.43	0.07	2.78
			c	5.0	0.4	5	0.12	6	-2.65	0.04	2.45
		50°C 28 d	b	4.2		1					
			c	5.3	0.3	4	0.08	5	-1.14	0.31	2.57
		70°C 14 d	b	4.1	0.1	2	0.01	3	-4.32	0.02	3.18
			c	5.0	0.3	5	0.10	6	-2.62	<0.04	2.45
		70°C 28 d	b	4.2	0.1	3	0.01	4	-2.60	0.06	2.78
			c	5.2	0.2	5	0.03	4	-2.18	0.10	2.78
		90°C 14 d	b	4.1	0.2	4	0.05	5	-2.38	0.06	2.57
			c	4.9	0.3	5	0.08	5	-2.92	0.03	2.57
		90°C 28 d	b	4.1		1					
			c	5.1	0.2	4	0.06	5	-2.24	0.07	2.57

Table S10: Coefficient of variation of the Ultem 9085 samples exposed to air. Type 4, 0/90° specimens and Type 4, -45/45° specimens are replaced by b and c in the table. The highest COV in each column is highlighted in yellow.

**Mechanical properties**	**E (%)**		**Yield strength (%)**		**UTS (%)**		**Strain at failure (%)**	
Samples	b	c	b	c	b	c	b	c
unaged	2.4	2.9	0.4	6.1	3.2	0.7	2.5	7.0
50°C 14 d	1.7	2.1	1.6	2.6	0.4	2.5	2.9	7.1
50°C 28 d		2.1		10.0		2.5		5.2
70°C 14 d	1.4	2.6	0.5	5.0	1.8	4.7	2.3	6.3
70°C 28 d	0.4	1.9	1.3	2.6	1.6	0.7	2.3	3.4
90°C 14 d	1.6	4.0	2.1	1.9	1.3	3.8	5.4	5.7
90°C 28 d		1.1		2.1		0.7		4.7
